# Regulatory miRNAs in Cardiovascular and Alzheimer’s Disease: A Focus on Copper

**DOI:** 10.3390/ijms23063327

**Published:** 2022-03-19

**Authors:** Anna Sacco, Fabio Martelli, Amit Pal, Claudia Saraceno, Luisa Benussi, Roberta Ghidoni, Mauro Rongioletti, Rosanna Squitti

**Affiliations:** 1 Department of Biology, University of Rome Tor Vergata, 00133 Rome, Italy; annasacco04@gmail.com; 2 Molecular Cardiology Laboratory, IRCCS-Policlinico San Donato, San Donato Milanese, 20097 Milan, Italy; fabio.martelli@grupposandonato.it; 3 Department of Biochemistry, AIIMS, Kalyani 741245, India; maximus1134@gmail.com; 4 Molecular Markers Laboratory, IRCCS Istituto Centro San Giovanni di Dio Fatebenefratelli, 25125 Brescia, Italy; csaraceno@fatebenefratelli.eu (C.S.); lbenussi@fatebenefratelli.eu (L.B.); rghidoni@fatebenefratelli.eu (R.G.); 5 Department of Laboratory Medicine, Research and Development Division, San Giovanni Calibita Fatebenefratelli Hospital, Isola Tiberina, 00186 Rome, Italy; maurociroantonio.rongioletti@fbf-isola.it

**Keywords:** Alzheimer’s disease, cardiovascular disease, microRNAs, copper, hypoxia

## Abstract

Non-coding RNAs (ncRNAs), including microRNAs (miRNAs), are key regulators of differentiation and development. In the cell, transcription factors regulate the production of miRNA in response to different external stimuli. Copper (Cu) is a heavy metal and an essential micronutrient with widespread industrial applications. It is involved in a number of vital biological processes encompassing respiration, blood cell line maturation, and immune responses. In recent years, the link between deregulation of miRNAs’ functionality and the development of various pathologies as well as cardiovascular diseases (CVDs) has been extensively studied. Alzheimer’s disease (AD) is the most common cause of dementia in the elderly with a complex disease etiology, and its link with Cu abnormalities is being increasingly studied. A direct interaction between COMMD1, a regulator of the Cu pathway, and hypoxia-inducible factor (HIF) HIF-1a does exist in ischemic injury, but little information has been collected on the role of Cu in hypoxia associated with AD thus far. The current review deals with this matter in an attempt to structurally discuss the link between miRNA expression and Cu dysregulation in AD and CVDs.

## 1. Introduction

Copper (Cu) is an essential transition metal present in traces in our body, and either a Cu deficiency or excess is life-threatening.

A number of studies have indicated that an imbalance in Cu levels and a high burden of oxidative stress are contributors to Alzheimer’s disease (AD), the main form of dementia in the elderly [1,2].

It is known that Cu is a cofactor in the regulation of a number of processes associated with hypoxia. This condition is triggered by an insufficient oxygen supply to tissues and organs that results from pathological conditions underlying AD and ischemic cardiovascular disease (CVD). Hypoxia response is mainly regulated by the hypoxia-inducible factor (HIF) family of oxygen-sensitive transcription factors [3], including HIF-1α, which is regulated by Cu [4].

HIF-1 regulates oxygen homeostasis by modulation of angiogenesis and vascular remodeling, as well as oxygen utilization. HIF also regulates glucose metabolism and redox homeostasis and plays a critical protective role in tissue response to hypoxia of ischemic heart disease, as well as myocardial infarction (MI) [5].

Under the condition of low oxygen levels, HIF-1 is not degraded but acts as a transcriptional target underlying several processes via hypoxia response elements (HREs) at the binding sites, defined as HBSs. Other factors modifying HIF-1′s ability to bind to HREs include epigenetic modifications such as CpG methylation and DNA damage caused by oxidative stress due to elevated intracellular levels of reactive oxygen species (ROS) [6].

The main pathological process triggering the onset of ischemic heart disease is an inflammatory process in which metabolic cells are activated, thereby increasing oxygen demand.

Cu is highly involved in inflammatory processes, either through ceruloplasmin, an acute phase reactant, or through non-ceruloplasmin Cu (also known as “free Cu”), since both increase during inflammation [7]. Despite the accumulated knowledge and technological achievements in amyloid beta (Aβ) and tau detection which build up in the AD brain and typify AD pathology and the new FDA-accelerated approval of the controversial disease-modifying drug Aducanumab (marketed as Aduhelm), which likely can reasonably translate to a clinical benefit [8], the current consensus in the field is that the cause of AD is incompletely defined. An alternative and valid approach toward AD should consider the disease as a multifactorial disorder where many risk factors contribute to global AD susceptibility. In this complex interplay, aging and oxidative stress—mainly through transition metal imbalance—constitute the leading risk factors [1,9]. Oxidative stress is a central molecular mechanism underlying transition metal-induced toxicity and hypoxia. During brain hypoxia, the autophagy machinery may abnormally accumulate high amounts of transition metals such as Cu and iron (Fe), which are normally present in the brain since they are necessary for correct brain functioning. When not bound to proteins or enzymes, Fe and Cu undergo redox cycling reactions with H_2_O_2_ (Fenton-type reactions), resulting in the production of ROS, of which H_2_O_2_ can diffuse through the cell membrane and then produce the very reactive hydroxyl radical (HO•) catalyzed by Cu and Fe (Fenton-type reactions) [10], eventually leading to tissue damage. The picture of Cu imbalance in AD emerging from a recent meta-analysis [11] is consistent with a shift or displacement of the metal from functional bound Cu to a labile toxic non-ceruloplasmin Cu pool that can easily cross the blood–brain barrier, affecting the aggregation of Aβ and likely producing oxidative stress [1,9].

Consistently, the literature has pointed at a brain-to-heart connection, focusing mainly on the associations between neurodegeneration and cerebral hypoperfusion, hypertension, genetic risk factors (*APOE4* and *MTHFR* gene mutations), high cholesterol, diabetes mellitus, obesity [12], and more recently Cu imbalance [13].

It is known that severe but also prolonged hypoxia is involved in AD neurodegeneration, as one third of stroke patients suffer from post-stroke dementia [14]. Despite this body of literature, the mechanisms linking AD and CVD—which may mediate heart failure (HF), MI, coronary artery disease, atrial fibrillation, and vasculopathy—are still not completely understood.

Among the newly identified mechanisms of Cu toxicity in AD, there is the loss of endothelial lipoprotein receptor-related protein 1 (LRP1), which has been shown to cause aberrant parenchymal Aβ buildup in various AD mouse models [15,16] mainly in a process that appears to be orchestrated by microRNAs [16]. As a matter of the fact, increasing evidence shows that hypoxia resulting from pathological conditions associated with AD and CVD induces the expression of a subset of microRNA (miRNAs) called hypoxia-induced miRNAs [3], which act as regulators of cell responses to a drop in oxygen tension.

miRNAs are short, single-stranded, non-coding RNA molecules of 19–25 nucleotides whose activity is crucial in gene silencing [17]. MiRNAs are an important epigenetic component, as miRNA expression is controlled by epigenetic modifications in a tissue-specific manner at the transcriptional level [18]. In the cytoplasm, miRNAs bind messenger RNAs (mRNAs) in a complementary way. This bond induces gene silencing through the inhibition of translation or degradation of mRNA [19]. One miRNA can influence the expression of hundreds of genes, and each mRNA molecule can be regulated by different miRNAs [20]. Most miRNAs are localized intracellularly but can be released into the blood’s circulation and travel to different districts, thus participating in cell–cell communication processes [21].

miRNAs are also present in the nucleus. Although the function of nuclear miRNAs has not been fully elucidated, the results of several studies show that nuclear miRNAs are involved in gene silencing and gene activation [22]. Most of the functions of miRNAs are linked to recognition by specific regions called miRNA response elements (MREs), which form the interaction link between miRNA and mRNA [23].

MiRNAs, together with ROS and other cell-signaling species such as NF-κB, a family of transcription factors, act as nodes of cross-talk in the inflammation cascade [24]. Several studies have revealed how specific miRNAs are regulated by HIF during hypoxic conditions, thus establishing a link between HIF, miRNA, and CVD states [25]. In the same line of thinking, since HIF is strictly linked to Cu regulation, HIF can be proposed as the possible missing link between Cu and miRNA regulation in AD, which is known to be associated with Cu imbalance [11] and cardiovascular risk.

In this review, several miRNAs involved in hypoxia processes underlying the pathophysiology states of AD and CVD are briefly discussed in the context of mechanisms of Cu toxicity, which is thought to be a driver of AD onset and progression [1].

## 2. Non-Coding RNAs and Cu Metabolism in Physiology

Non-coding RNAs (ncRNAs) are key regulators of differentiation and development. In 2003, the Human Genome Project (HGP) was completed, and about 20,000–25,000 genes were identified, offering the opportunity to predict the outcomes of individuals diagnosed or threatened with complex diseases such as AD [26].

Cu is a heavy metal and an essential micronutrient with widespread industrial applications. As an essential metal, it is involved in a number of vital biological processes encompassing respiration, blood cell line maturation, immune responses, wound healing, myelin sheath formation, and neurotransmitter synthesis and regulation. It is indispensable for brain and heart development and correct physiology. Severe Cu deficiencies can cause cardiac, bone, immune, and central nervous system conditions, while Cu chronic excess, exposure, or displacements in tissues and organs are associated with liver damage and neurodegenerative disorders [27].

The interplay between miRNA regulation and Cu in physiology will be discussed herein.

### 2.1. Role of microRNAs in Cell Regulation in Physiology

The genome consists of a large fraction of sequences of RNAs that do not encode any proteins [28] according to Palazzo and collaborators. It is estimated that 99% of the total RNA species present in mammalian cells are non-coding RNAs [29].

The ncRNAs can be classified by length (small: 18–200 nt; long: >200 nt) [30] or by function. The housekeeping ncRNAs include tRNAs and rRNAs, while the regulatory transcripts comprise miRNAs [31]. The miRNAs are a group of small ncRNA molecules.

The biogenesis of miRNAs involves being encoded by genomic DNA with the action of RNA polymerase II, which generates the primary RNA (pri-miRNA). This first product is usually several kilobases long. Then, the pri-miRNA is processed by RNase III Drosha into short hairpin structures called pre-miRNAs, which are exported from the nucleus by exportin. In the cytoplasm, the RNase III enzyme Dicer cuts the pre-miRNAs into mature miRNAs composed of about 22 bases [26]. In association with the Argonaute (AGO) proteins, miRNAs form the RNA-induced silencing complex (RISC), which allows for the translational repression or degradation of the target mRNA. The action of the miRNA on the mRNA depends on the complementarity of the miRNA with its target. When the complementarity is imperfect, the result is translational repression [32], but target RNA destabilization is also observed very often.

According to recent studies, the regulation of miRNA functionality and expression is controlled at three different levels: transcription, processing, and subcellular localization.

In the cell, the transcription factors regulate the production of miRNAs in response to different external stimuli such as inflammation. This process permits control at the transcriptional level [33].

There are several regulatory mechanisms that control miRNA maturation at various stages, starting from the primary transcript. Some of these factors, are the SMAD proteins [34] and Arsenate-resistance protein 2 (ARS2), which permit processing of pri-miRNA [35]. In addition, the tumor suppressor protein p53 has an important role in miRNA processing. The link between p53 and the protein Drosha increases the processing of pri-miRNA to pre-miRNA, and this interaction induces the maturation of specific miRNAs. The levels of p53 in turn can be negatively or positively regulated by specific miRNAs [36,37].

The processing of miRNAs can be post-transcriptionally regulated by RNA-binding proteins (RBPs), which can recognize some regions of miRNA precursors and modulate the processing efficiency. One of these regulators is the stem cell factor LIN28, which interacts with the pre-miRNAs and blocks their expression [38]. Another regulatory factor is HNRNPA1, which binds to pri-miR-18a and increases the function of Drosha [39]. Nussbacher and collaborators found that 92% of RBPs interact directly with one miRNA locus, and they are cell line specific [40].

miRNA is the most studied class of ncRNA. miRNAs recognize their targets through miRNA response elements (MREs). Generally, the most conserved binding sites are enriched in the 3′UTR sequences, but MREs can also be present in the 5′ UTR sequences as well as in the coding regions of mRNAs [41]. miRNAs form a complex (RISC) by binding to various AGO proteins, allowing the miRNAs to recognize their targets and to bind and detach easily from targets. Furthermore, AGO proteins are important cofactors for the binding of RISC with the target, and different types of AGO proteins mediate different gene regulation [42].

In the cytoplasm, one of the primary functions of the miRNA-RISC (miRISC) is post-transcriptional mRNA regulation [43]. Cytoplasmic nuclear shuttling is a studied function of miRISC [44,45,46]. miRNAs have many nuclear functions, and some of these are independent of RISC activity.

miRISC can regulate non coding transcripts, too. Indeed, this complex can target lncRNAs, modifying their stability and function [27,28,29].

### 2.2. Cu Intake in Human Diet and Drinking Water

**Figure 1:**
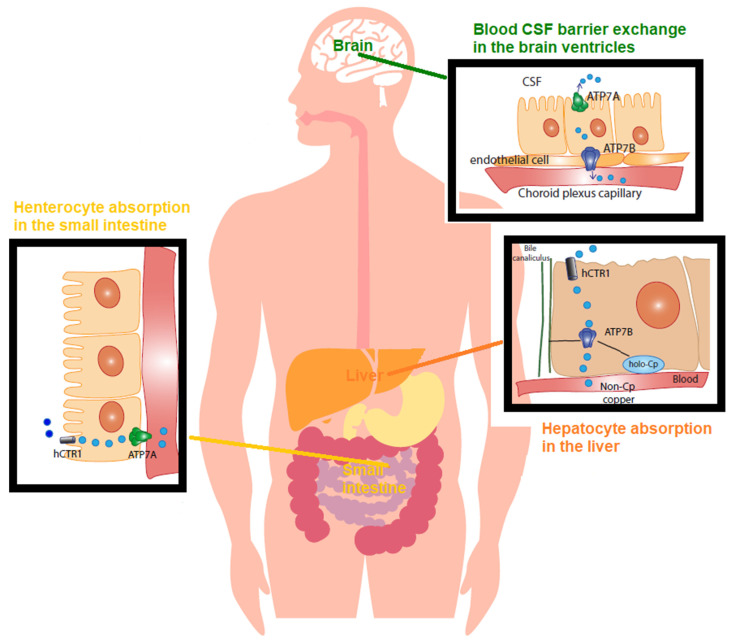
Copper (Cu) in physiology The recommended dietary allowance (RDA) for Cu is 0.9–1.3 mg/day. This represents the intake level sufficient to meet the nutrient requirements. Accordingly, humans normally ingest 1.5 mg/day of Cu via beverage and foods. The Cu balance is determined by the equilibrium between the rates of dietary absorption from diet and excretion through stools and bile, and it is tightly regulated. In the small bowel, concerning Cu absorption into the enterocyte, Cu^2+^ is reduced by reductases. CTR1 imports Cu^1+^ within the cell. Cu is absorbed as a pool of low molecular weight soluble complexes and pumped out of the enterocyte’s basolateral membrane by the Cu-transporting P-type ATPase (ATPase7A) via the vesicular compartment (not shown). Cu is then transported, mostly bound to amino acids, peptides, micronutrients, and albumin, and transported into the serum. From the gut, this pool of low molecular weight Cu, known as non-ceruloplasmin (non-Cp) Cu, travels to the liver through the portal vein. The liver represents the main organ of storage and utilization of Cu. CTR1 facilitates Cu intake in the hepatocytes and delivery to chaperones. In the liver, ATPase7B, the homologue of enterocytes’ ATPase7A, incorporates Cu into ceruloplasmin. Of the Cu, 75–95% tightly binds to ceruloplasmin, whereas the remainder loosely binds to and is exchanged among albumin, α2 macroglobulin, amino acids, peptides, and several micronutrients. Hepatocytes limit the non-Cp Cu concentration in the blood to 0.008–1.6 µmol/L (equivalent to 0.05–1 mg/dL), which is the upper limit of the normal reference range of non-Cp Cu in serum after an overnight fast. An excess of Cu induces the translocation of ATPase7B from the trans-Golgi network to the canalicular membrane (via the vesicular compartment), where the metal is released into the bile. As for blood barrier exchange in the brain ventricles, the endothelial cells of the brain’s capillaries constitute the blood–brain barrier (BBB). The cerebrospinal fluid (CSF) is the biological fluid that surrounds the brain and fills the brain ventricles, and it is secreted by the choroid plexus. The Cu in CSF has values in the range of 0.5–2.5 µmol/L. In the choroid plexus, non-Cp Cu is the main form of Cu taken up from the blood and is then released into the brain by processes mediated by CTR1, ATPase7A, and ATPase7AB.

Cu balance is established by the rates of dietary absorption from food, supplements, drinking water, and excretion through stools and bile, and it is tightly controlled (Figure 1).

The Cu recommended dietary allowance (RDA)—the intake level sufficient to meet the nutrient requirements for 97–98% of people—is 0.9–1.3 mg/day (United States (US)) [47]. On average, the individual consumes ~2 mg per day (WHO, 1996) [48], and 2–3 mg/day of Cu intake is safe and adequately prevents Cu deficiency, while ingesting >5 mg/day is deemed toxic [49].

### 2.3. Cu Regulation in Physiology

The Cu from a diet is readily absorbed from the stomach and intestines (duodenum and ileum) at a rate of 0.5 mg/day [50]. Then, it is transported to the liver (Figure 1), and 0.5 mg/day generally corresponds to 30% of the intake (75% from food and 25% from supplements and beverages). The remaining amount of Cu is directly eliminated through the “mucosal block”, consisting of metallothioneins (MTs), which are cysteine-rich proteins that bind and sequester metal ions and are located in the enterocyte cells lining the mucosal tract, which trap Cu and facilitate its excretion [50]. The rate of Cu absorption lessens as the Cu intake rises. The dietary Cu intake changes from 56% during a low-Cu regimen to 12% under a high-Cu diet [51]. Even though in a high-Cu intake diet the efficiency of Cu absorption declines, more Cu is absorbed and retained [51].

In the stomach, duodenum, and ileum, Cu is absorbed as a pool of low molecular-weight-soluble complexes of Cu [52]. In the intestinal lining cells, Cu is then pumped out of the enterocyte by the Cu-transporting P-type ATPase (Cu-ATPase) 7A (ATPase7A) located at the basolateral membrane. This pool of Cu is called non-ceruloplasmin Cu, a serum Cu^2+^ species not structurally bound to proteins and primarily not bound to ceruloplasmin, the main Cu protein in general circulation [53]. Non-ceruloplasmin Cu is mostly loosely bound to low molecular weight compounds such as amino acids, peptides, and micronutrients, and it is exchanged among them, albumin, and α2-macroglobulin and transported into the serum. From the gut, it reaches the liver through the portal vein. In physiology, hepatocytes take up most of this Cu species and regulate the blood’s non-ceruloplasmin Cu concentration in the range of 0.008–1.6 µmol/L (equivalent to 0.05–1 mg/dL). These are the normal reference range values of non-ceruloplasmin Cu in serum after an overnight fast [50,54] (Figure 1).

In the liver, namely in the interstitial fluid surrounding cells, human Cu transporter 1 (CTR1) regulates Cu(I) entry into the hepatocyte, and Cu chaperons and transporters then accompany the metal to the sites of its utilization within the cell [55]. At the trans-Golgi network, Cu is packed into Cu proteins and enzymes. The main pathways include cytochromes in the mitochondria for oxidative respiration (cytochrome oxidase (COX)), copper/zinc superoxide dismutase (Cu/Zn SOD) for antioxidant defenses, and ceruloplasmin for controlling the iron (Fe) oxidative state [55].

Similar to ATPase7A, the Cu-transporting P-type ATPase (Cu-ATPase) 7B (ATPase7B) is a key protein controlling the Cu balance. It is a Cu pump located in the trans-Golgi network that loads Cu into nascent ceruloplasmin in the hepatocyte [55] and facilitates Cu excretion into the bile when the metal exceeds the needs of the cell [50] (Figure 1). Ceruloplasmin binds 75–85% of circulating Cu, whereas the remainder constitutes the non-ceruloplasmin Cu species [55].

In correct physiology, Cu in the CSF, the biological fluid that surrounds the brain and is produced and secreted by the choroid plexus, ranges between 0.5 and 2.5 µmol/L [56]. Perfusion of three species of radioactive ^64^Cu (a homolog of non-ceruloplasmin ^64^Cu, ^64^Cu-albumin, and ^64^Cu-ceruloplasmin) into a rat brain via the internal carotid artery demonstrated that non-ceruloplasmin ^64^Cu in a rat choroid plexus is the main species taken up into the brain, being about 50 times higher than Cu-albumin and 1000 times higher than Cu-ceruloplasmin [57]. This is also exemplified in Wilson’s disease, the paradigmatic disorder for non-ceruloplasmin Cu toxicosis or accumulation, in which non-ceruloplasmin Cu easily crosses the blood–brain barrier (BBB) [50], and it has also been found in AD [58].

Wilson’s disease is a rare genetic disorder caused by mutations in the *ATP7B* gene, encoding for the ATPase7B the Cu pump that handles Cu incorporation into ceruloplasmin and Cu excretion through the bile. *ATP7B* mutations cause Cu accumulation in the liver, kidneys, and other parenchymal tissues, including the brain [59]. Cu deposition in the cornea and around the iris of the eye, known as Kayser–Fleischer rings (for a specialized review, refer to [60]), is typical. Wilson’s disease is also typified by levels of non-ceruloplasmin Cu higher than the 1.6 µmol/L that is considered toxic [61]. Aside from eating food and supplements and drinking water, Cu exposure can also occur through breathing air or by skin contact with soil, water, and other Cu-containing substances, including nanoparticles [62].

## 3. MicroRNA and Cu Metabolism in Disease States

### 3.1. Cardiovascular Diseases

Cardiovascular disease is a class of diseases that embraces coronary heart disease, including MI (or angina pectoris), cerebrovascular disease (spanning from stroke to transient ischemic attack), high blood pressure, peripheral artery disease, heart failure, abnormal heart rhythm, and congenital heart disease [63]. It represents the largest cause of death worldwide in developed countries, causing about one third of all deaths in individuals over 35 [63]. Improving risk factors can prevent up to 90% of CVD.

Atherosclerosis and coronary heart disease are characterized by inflammation and oxidative stress.

Cu and ceruloplasmin are central drivers of oxidative stress and inflammation [7]. Specifically, ceruloplasmin is an acute phase reactant and increases in inflammation states [7]. Recently, it evidence has been provided that supports a direct relationship between elevated ceruloplasmin levels and CVD. Individuals with high ceruloplasmin serum concentrations are more likely to have CVD [64]. Most of the studies analyzed in a recent systemic review by Arenas de Larriva and collaborators [64] showed a direct relationship between serum ceruloplasmin levels and the incidence of CVD. The higher the patient’s serum ceruloplasmin level, the more likely the patient is to experience CVD complications.

The mechanism by which ceruloplasmin and Cu may influence CVDs is still partially elusive. Recent studies have shown that Cu, and specifically the Cu/zinc ratio [65], is strongly associated with the indices of inflammation. The primary role of ceruloplasmin is to scavenge ROS, such as superoxide and hydrogen peroxide, so it may be relevant in the majority of biological mechanisms underlying CVD. It is hypothesized that with high levels of ROS, antioxidant systems such as Cu/Zn SOD, catalase, and glutathione are overwhelmed, and the structural integrity of ceruloplasmin is impaired [64].

Ceruloplasmin is involved in the removal of Fe from cells, and its dysfunction, particularly its loss of ferroxidase activity, can lead to Fe accumulation in tissues, which is a potential source of ROS. In addition, ceruloplasmin loss of function produces non-ceruloplasmin Cu which, together with Fe, can produce pathogenic effects in the cell such as apoptosis, cell toxicity, cell replication, increased oxidative stress (via Fenton-type reactions), and pathogenic gene activation. Non-ceruloplasmin Cu is a marker of Wilson’s disease, and it has been recently advocated as a stratification marker of the Cu AD subtype (CuAD) [1,66]. It is reasonable to argue that increased levels of non-ceruloplasmin Cu in CuAD and CVD would trigger similar downstream pathological processes. The findings from a mouse model of cardiomyopathy showed the link of myocardial hypoxia, increased activity of the Cu transporter COMMD1 (MURR domain 1), and non-ceruloplasmin Cu (Li and collaborators 2018) [67]. In such a condition, activation of HIF may likely occur. As a matter of fact, during inflammation, tissues are often hypoxic, and in the cellular response to low oxygen tension, HIF-1 orchestrates a metabolic switch that allows cells to survive in this environment [68].

### 3.2. Involvement of miRNA in Cardiovascular Disease

The human transcriptome comprises a network of coding and ncRNAs involved in different biological functions. In recent years, a number of different studies have been conducted on the association between cardiovascular disease and miRNA regulation.

A number of studies have demonstrated that miRNAs can be significant for diagnosis and treatment in HF, because cardiomyopathy, hypertension, diabetes, and other causes are associated with several miRNAs.

Prasad and collaborators [69] showed that some miRNAs revealed by RT-qPCR and a microarray were increased in dilated cardiomyopathy (DCM compared with controls), suggesting a potential role of miRNAs as biomarkers of HF [69].

Other studies revealed that the expression of miR-454 and miR-518f was higher in pediatric patients affected by idiopathic DCM compared with the controls [70].

Zeng and collaborators [71] demonstrated that the plasma miR-182 level was negatively associated with the left ventricular ejection fraction (LVEF) and positively associated with HIF-1 in a group of patients with ischemic cardiomyopathy (ICM) compared with the controls. LVEF and HIF-1 are essential for the understanding of the severity of a cardiac disease. Thus, miR-182 may be thought to reflect the severity of a disease [71].

In another study, Olsen observed the upregulation of miR-208 and miR-499 along with the downregulation of miR-126, miR-17, and miR-92 in patients with coronary artery disease [72]. In 2016, Li and collaborators [73] observed that 55 miRNAs were increased in ischemic cardiomyopathy (ICM) and in non-ischemic cardiomyopathy (NICM) and that 38 miRNAs were downregulated in both ICM and NICM. The results of the study suggest that miR-182 could be employed as a diagnostic biomarker for ICM and DCM [73].

miRNAs are associated with hypertension, which is a risk factor for cardiac and vascular disease and AD since long-term hypertension can induce myocardial cell hypertrophy, the primary cause of HF [74]. As per several studies, miR-208 may be the cause of cardiac hypertrophy through overexpression of β-myosin [75]. Other results demonstrate that some significant factors are mediated by miRNAs. For example, insulin-like growth factor (IGF)-1 is mediated by miR-1, indicating that miR-1 may be a diagnostic and therapeutic CVD biomarker [76].

The transcriptomes, genomes, and epigenomes from individual cells and cell types in complex heterogeneous organs and tissues of the CV are described through DNA and RNA technical sequencing. The miRNA-1254 levels predicted changes in the left ventricular (LV) volumes and LV ejection fraction 6 months after ST elevation MI (STEMI) [77].

Several sources in the scientific literature demonstrated that miRNAs are essential to regulate postnatal cardiomyocyte proliferation and cell regeneration. Therefore, miRNAs not only affect cardiovascular pathologies but are also fundamental for the generation of cardiomyocytes in the embryonic development phase and for the regeneration of cardiac cells after harmful events such as HF. Eulalio and collaborators [78] demonstrated that miR-590 and miR-199a promote cell cycle reentry and the proliferation of adult cardiomyocytes [78]. Furthermore, mouse miR-302–367 cluster activation appears to induce cardiomyocyte proliferation and heart tissue regeneration after HF [79]. In contrast, in the adult mouse heart, miR-15 family inhibition from the early postnatal stage induces the regeneration of the organ [80].

The hypoxic process occurs under many circumstances. It may be due to tissue growth, organ development, tumor expansion, or ischemic damage. Furthermore, hypoxia is also caused by high altitudes [81]. Generally, the cells damaged by hypoxia are eliminated from the tissue and replaced through the implementation of complex molecular processes. In the last few years, several studies demonstrated that a neonatal heart is capable of regenerating lost myocardium [82], while an adult heart is capable of retreading only small regions of tissue [83].

Fasanaro and collaborators [84] described the substantial role of miRNAs in endothelial cells’ (ECs) response to hypoxia. Particularly, they showed that miR-210 was a driver of ECs’ response to hypoxia. In this study, the authors quantified miRNA and mRNA in HUVECs and evaluated the hypoxia effect on miR-210 expression, exposing them to low oxygen concentrations for different periods of time. The results demonstrated that the miR-210 levels increased in the ECs undergoing hypoxia. The hypoxic process caused oxidative stress and acidosis, and these conditions were sufficient to increase the miR-210 levels, as this overexpression was directly proportional to the hypoxia. Furthermore, hypoxia caused an increase in capillary-like structure formation and in EC migration. Interestingly, miR-210 blocking inhibited EC tubulogenesis and migration. miR-210 directly targets and inhibits ephrin A3 (EFNA3), a receptor tyrosine kinase family essential for migration, adhesion, and repulsion during neuronal, vascular, and epithelial development [84]. miR-210 modulates different gene pathways, and for this reason, it can potentially have two different and even opposite effects, depending on the physio-pathological context [85].

Several studies have associated differential expression of various miRNAs in response to HIF-1, where miR-210, miR-155, miR-372/373, and miR-10b are upregulated during hypoxia [86,87] while miR-20b and miR-200b are downregulated [88,89]. However, the results for the miRNAs involved in MI are controversial, since some miRNAs are upregulated in some studies and downregulated in others. These results are probably due to the heterogeneity of the clinical manifestations associated with MI and mainly due to the number of miRNA subtypes belonging to the same family [90]. The results of these studies support the idea that in the adult mammalian heart, miRNAs have some potential as therapeutics [91,92].

miRNAs have various limitations as a biomarker of HF. First, it is difficult to assess which mechanisms initiate changes in circulating miRNA levels because various molecular pathways overlap.

In addition, the levels of miRNAs in serum or plasma are generally low to the point that they are hardly detectable. Furthermore, the literature reports controversial results on the miRNA levels in patients with HF following treatment [93,94].

### 3.3. Brief Description of Alzheimer’s Disease’s Main Features

AD is a complex disease and the most common cause of dementia in the elderly, being defined as a group of symptoms [95]. In 2019, the people affected by AD approximated 50 million. As the size of the older population grows, the number of living individuals with AD is expected to reach more than 152 million by 2050. In 2019, about USD 1 trillion was spent on AD patient care [95].

As per the World Health Organization (WHO), the top seven countries in the number of deaths caused by AD are China (563.5), USA (259.5), India (140.9), the United Kingdom (82.5), Indonesia (54.7), France (49.6), and Germany (48.8) in terms of thousand deaths in 2016 [96].

AD is distinguished by an early (<65 years old) or late (>65 years old) onset and is normally classified as familial (<5%) and sporadic with a multifactorial disease etiology. The early-onset familial form is detected by the presence of gene mutations in *APP*, *Presenilin1*, or *Presenilin2* [97].

Aging, family history, and the *APOE4* gene are the main risk factors for late-onset AD [96]. The WHO [97] has provided recommendations to lower the risk of cognitive decline and dementia by increasing physical activity, quitting smoking, and handling cardiovascular risk factors (mainly diabetes, obesity, smoking, and hypertension). Implementing a healthy diet, lifelong learning, and cognitive training have been associated with a reduced risk of cognitive decline in agreement with previous expert committee recommendations [98,99].

AD is an irreversible neurodegenerative disorder characterized by a gradual loss of cognition, eventually leading to dementia and death. The 2018 National Institute on Aging—Alzheimer’s Association (NIA-AA) Research Framework [100] represented the cognitive continuum as a syndromic cognitive staging split in the three categories: cognitively unimpaired, mild cognitive impairment (MCI), and dementia. Dementia can be further stratified into mild, moderate, and severe stages. Dementia from an early stage is characterized by a mild loss of recent memory. In a period of 8–10 years after diagnosis, the disease advances to loss of faulty judgments and personality changes, terminating in a complete loss of reasoning ability and self-sufficiency.

The AD brain is featured by atrophy, consisting of dilated ventricles, narrower gyri, and sulci wider than those in a normal brain. The loss of tissue is severe, and an AD brain can weigh less than 1 kg. The hippocampus is severely affected, followed by the frontal, parietal, and temporal lobes. The primary sensory-motor cortex is affected in the later stages, while the occipital lobe is generally spared.

The amyloid plaques, consisting of the accumulation of the protein fragment beta-amyloid (Aβ) outside neurons and twisted strands of the protein tau (neurofibrillary tangles) inside neurons, are pathologic hallmarks of the AD brain. Amyloid plaques are brain formations made up by clusters of protein fibrils around an amyloidal core, abnormal neurites, and glial cells. The aggregation of Aβ peptides, which are produced by the proteolytic cleavage of the amyloid precursor protein (APP), constitute mainly the amyloid. Generally, APP is cleaved on the extracellular side of the neuronal membrane by the enzyme α-secretase, but it can be cleaved by two other enzymes (β- and γ-secretase) at different sites, resulting in the production of Aβ segments that are released in the extracellular space and aggregate there in amyloidal plaques.

Neurofibrillary tangles consist of aggregates of the hyper-phosphorylated protein tau in the intracellular space. In normal physiology, the protein tau binds to microtubules and contributes to neuronal cytoskeleton stability. In AD, tau protein undergoes hyper-phosphorylation, which causes the protein to aggregate and the loss of its binding capability to microtubules, which disintegrate.

In the AD brain, the areas most affected by neurofibrillary tangles are the hippocampus, subiculum, amygdala, and entorhinal cortices, and numerous neurofibrillary tangles can also be found in the nucleus basalis, limbic nuclei of the thalamus, locus coeruleus, substantia nigra, and the raphe nuclei of the brainstem.

miRNAs have been the focus of recent research in AD because of their potential as new putative diagnostic and therapeutic tools. A recent meta-analysis showed that 40 miRNAs were significantly upregulated and 16 were downregulated in AD versus healthy controls. The analysis revealed that they were mainly involved in pathways related to apoptosis, immune response, and inflammation [101].

### 3.4. Involvement of Cu in the Induction of MicroRNA in Alzheimer’s Disease

Cu imbalance has been linked to AD risk and pathogenesis [1,11,102]. The effects of Cu toxicity are thought to be associated with abnormal energy metabolism, an increase in glycolysis, and mitochondrial oxidative and DNA damage, as recently structured in a disease hypothesis construct [103]. In 2013, Singh resumed Sparks Schrewer’s seminal studies on AD and demonstrated that excess non-ceruloplasmin Cu is toxic [104]. (Readers are referred to specialized articles for the non-ceruloplasmin Cu role in AD [1,11].) In that study, Singh and collaborators (2013) showed that 0.13 mg/day of Cu in drinking water for 90 days induced Cu accumulation in the brain capillaries of aging mice and reduced the levels of low-density lipoprotein receptor-related protein 1 (LRP1). LRP1 is involved in clearance of Aβ, and LRP1 dysfunction is thought to facilitate Aβ accumulation in the brain, eventually leading to the formation of amyloid plaques [15].

More recently, Hsu and collaborators [16] demonstrated that Cu decreases LRP1 expression. They examined the temporal changes of LRP1 expression, in which human primary brain microvascular endothelial cells (MVECs) treated with Cu had reduced levels of LRP1 after 24 h and continued to show LRP1 downregulation even after 24 h of recovery. The MVECs returned to the basal values at 48 h from treatment with Cu when the levels of LRP1 began to increase. It is noteworthy that the investigation of the mechanism underlying LRP1 downregulation induced by Cu exposure led to the discovery of several miRNAs involved in LRP1 Aβ regulation; miRNA-205-5p, miRNA-200b-3p, and miRNA-200c-3p were specifically overexpressed in the MVECs treated with Cu, and they repressed LRP1 translation [16]. The authors demonstrated that LRP1 downregulation was associated with an increase in Aβ brain vascular accumulation and with a loss of spatial memory in the Cu-treated mice. In line with this study, Yu and collaborators showed that a low-dose Cu treatment in mice can induce neuronal cell apoptosis in the hippocampus. When analyzing the proteomic profile of these cells, it emerged that the mitochondrial proteins were differentially expressed in the treated and control mice. Mitochondrial protein differential expression has effects on cell viability. More specifically, bioinformatics analysis revealed that 31 mitochondrial proteins and 46 nucleoproteins were differentially expressed after Cu exposure and that these proteins were involved in synaptic dysfunction, DNA damage, apoptosis, energy metabolism, and oxidative stress. As a whole, these alterations induce synaptic dysfunction and neurotoxicity [105].

Of relevance is that Magenta and collaborators examined the effect of ROS on microRNA (miRNA) expression in EC exposed to 200 mM hydrogen peroxide in vitro and in skeletal muscle following acute hindlimb ischemia in a mouse experimental model. miRNA profiling showed that miR-200c and the co-transcribed miR-141 increased more than eightfold, inducing HUVEC growth arrest, apoptosis, and senescence. miR-200c targets ZEB1 mRNA, and accordingly, the ZEB1 protein was downmodulated by miR-200c overexpression [106].

However, Liu and collaborators demonstrated that miR-200b was downregulated in AD [107], while other groups have shown upregulation [108]. Non-univocal data may be ascribed to the different biological matrices analyzed, as in the first study cited, the miRNAs were extracted from hippocampal neural cells and were therefore cellular miRNAs. In the second study cited, the circulating miRNAs in blood and cerebrospinal fluid were assessed [107,108]. In fact, intracellular and extracellular miRNAs can often display opposite regulations.

These discordant results suggest that other studies should be performed to clarify the evidence linking Cu exposure to miRNA regulation in AD.

### 3.5. Involvement of Cu in Myocardial Infarction

Recently, the dynamics of Cu dysfunction triggered by MI onset have been defined in a series of refined experiments. In mouse and rat models of experimentally induced ischemic MI induced by the ligation of the left anterior descending (LAD) coronary artery, some authors showed that myocardial Cu is released into the bloodstream by the increased activity of the Cu transporter COMMD1 (MURR domain 1) [109,110]. In another study by Ying Xiao Chen Li and collaborators [111], the authors reconstructed the different phases of Cu release in the bloodstream caused by MI. In this study, adult male Sprague Dawley rats underwent LAD, and the serum Cu and ceruloplasmin levels, as well as changes in the hepatic ceruloplasmin and Cu-transporting ATPase7B (ATPase7B) levels, were measured in blood and liver samples collected on days 1, 4, or 7 after surgery. The serum Cu concentrations increased significantly on day 4 after LAD ligation, accompanied by an increase in the serum levels of ceruloplasmin concentration and activity. At the same time, the ceruloplasmin and ATPase7B levels significantly increased in the liver. Furthermore, inhibition of the increase in serum Cu by employing the Cu chelator triethylenetetramine effectively abolished the elevated ceruloplasmin activity after LAD ligation. These results support the notion that the increase in serum ceruloplasmin in response to MI is most likely the result of increased hepatic ceruloplasmin production, which in turn is activated by increased serum Cu released from the injured myocardium, rather than being consequent to an inflammatory response.

### 3.6. Are the miRNAs Induced by Hypoxia, the Missing Piece between Cu and Cardiovascular Disease?

The incessant activity of the heart requires a continuous supply of energy (ATP). On an empty stomach, 60–70% of cardiac metabolism is related to fatty acid metabolism, but after a meal, the blood levels of insulin increase and induce a change in cardiac metabolism, which starts to consume glucose. This mechanism ensures that the heart is always supplied with appropriate energy sources [112]. In the heart, ATP is provided by oxidative phosphorylation reactions in mitochondria in processes that need oxygen, and chronic hypoxia induces cardiac vessel fatty acid metabolic remodeling [113]. Hypoxic conditions induce the expression of HIF-1α, which plays an important role in reprogramming myocardial metabolism. Indeed, HIF-1 signaling in endothelial cells results in improved ventricular function and size, allowing cell survival and tissue reperfusion [114,115].

In the patients at high risk of CVD, HIF-1 levels increase significantly compared with the control groups, as well as the Lp-PLA2 level, which is a marker of inflammation, the first stage of the atherosclerotic process [116] that is strongly connected with Cu regulation.

Aside from inflammation, chronic or severe hypoxia in AD can affect ATP production, calcium homeostasis, and oxidative stress but can mostly stimulate the amyloidogenic Aβ cleavage of APP. The facilitating Aβ plaque aggregates formation via β-secretase 1 (BACE1). The BACE1 DNA promoter binds to HIF-1, and HIF-1α overexpression in neurons induces BACE1 mRNA and protein synthesis (for a specialized review, refer to [117]). It is known that BACE1 has a transmembrane Cu-binding domain, and overexpression of BACE1 can lead to reduced Cu/Zn SOD activity and amyloidogenic Aβ cleavage [118].

**Figure 2:**
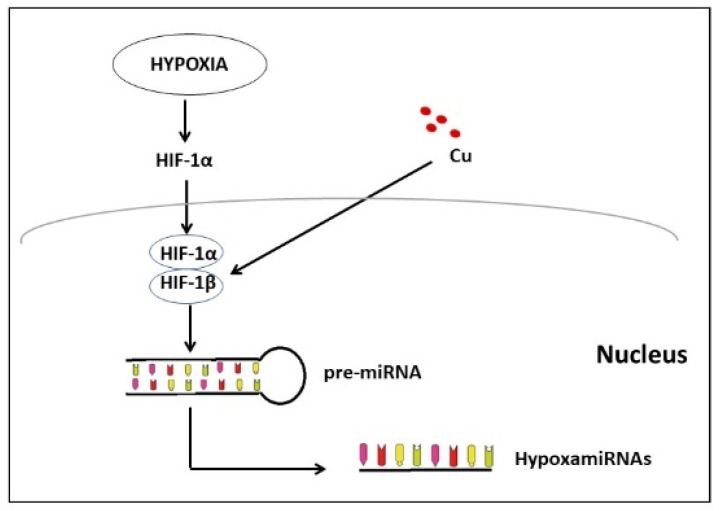
Hypoxia-induced miRNAs. Hypoxia-inducible factor 1 (HIF-1) mediates hypoxia response. The activation of many O_2_-regulated genes is mediated by HIF-1, a heterodimer consisting of HIF-1α and HIF-1β. HIF-1α stimulates glycolytic gene expression in different types of cells and induces the angiogenesis process. Increased Cu levels induce stabilization of the HIF-1α subunit, and HIF-1α binds HIF-1β and forms a heterodimer that migrates into the nucleus, where transcription of hypoxia-related genes starts. In addition to the activation of gene transcription, the role of HIF-1 in pre-miRNAs and inducing the maturation of hypoxia-related miRNAs is also hypothesized.

It could be hypothesized that there is a link between Cu dysfunction following CVD and the induction of miRNAs linked to CVD induced by hypoxia (Figure 2). Hypoxia is a crucial component of ischemic CVD. Hypoxia induces molecular changes and triggers the transcription of HIFs [119]. HIFs consist of two basic protein subunits: α (HIF-1α or HIF-2α) and β (HIF-1β or ANRT). In the conditions of adequate oxygen perfusion, HIF-α subunits are rapidly degraded by the ubiquitin–proteasome pathway, while in hypoxic conditions, the HIF-α protein remains stable and forms a complex with HIF-β [120].

In addition to causing degradation or stabilization of the HIF-α and HIF-β heterodimer, hypoxia regulates the expression of approximately 50 specific miRNAs named hypoxia-induced miRNAs or hypoxamiRs.

Over the years, within this group of miRNAs, miR-210 has been identified as closely related to the response to hypoxia binding different target genes [121].

Cicchillitti and collaborators [122] demonstrated the specific HIF-1α binding site for miR-210 promoter activation [123]. Furthermore, miR-210 modulates mitochondrial metabolism [122].

Li-Li Zeng and collaborators [124] studied the miRNA profiles in ischemic brain injury, and improved neuronal repairing and remodeling was crucial and timely. The miRNAs showed distinct expression patterns that modulate pathogenic processes including atherosclerosis, hyperlipidemia, hypertension, and plaque rupture. This paper shows that Lentivirus-mediated miR-210 overexpression enhanced the microvessel density and the number of neural progenitor cells in the ischemic mouse brain.

Mutharasan and collaborators [77,125], demonstrated that miR-210 is upregulated through both HIF-dependent and independent pathways. In the HIF-1β knockout mouse, protein kinase B (AKT) increased the miR-210 levels under hypoxia. HIF inhibition induced a decrease in miR-210, while the AKT overexpression resulted in increased levels of miR-210 in HIF- β knockout mice as well. This pathway has an important role in protection against oxidative stress [125].

As seen previously, Hsu and collaborators showed that the Cu levels regulate the expression of different miRNA targets. Cu loss is associated with an upregulation of COMMD1 domain 1 in the heart [109], but COMMD1 function in the heart is not clear [126]. Cu’s presence allows HIF-1 transcriptional complex formation, which binds to the HRE sequence of angiogenic genes [127,128].

Several studies have clarified that certain transition metals, including Cu, improve the stabilization of the HIF-1 protein. Excess Cu can stabilize HIF-1 through the inhibition of prolyl hydroxylase [129]. In addition, Cu is essential for the transcriptional activity of HIF-1, as the absence of Cu in cell cultures induces the blockade of insulin-like growth factor-1 (IGF-1)-induced HIF-1 and is linked to the HRE and vascular endothelial growth factor (VEGF) expression. The synthesis of, stabilization of, translocation from the cytosol to the nucleus of, binding to the HRE sequence of target genes of, and formation of the HIF-1 transcriptional complex all require the presence of Cu at the cellular level [129]. Cultured liver cells treated with TEPA, a Cu chelator, show how VEGF production decreases in the absence of Cu and how a higher Cu concentration causes VEGF production to recover under hypoxia conditions. At the same time, the HIF-1 levels were measured by a luciferase assay, and the results showed a decrease in the HIF-1 levels in the absence of Cu [4].

**Figure 3:**
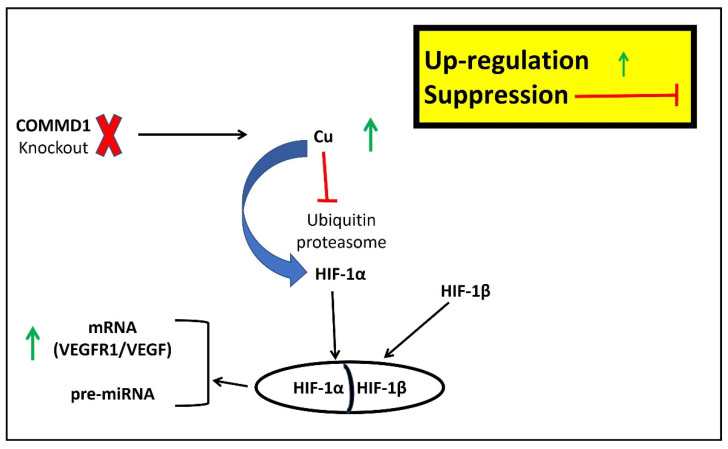
Hypothetical model of HIF-1 regulation by COMMD1. COMMD1 knockout prevents Cu loss in the cardiomyocyte after myocardial infarction (MI). Increased Cu levels induce HIF-1α stabilization and HIF-1 migration in the nucleus. Cu stabilizes HIF-1 through inhibition of the ubiquitin-proteosome complex which, under normal oxygenation, degrades the HIF-1α subunit and prevents formation of the HIF-1α/HIF-1β heterodimer. Cu might have a dual action on HIF-1, as it acts on the formation of the HIF-1α/HIF-1β heterodimer but also on its transcriptional activity, because Cu’s absence blocks insulin-like growth factor-1 (IGF-1) and vascular endothelial growth factor (VEGF) expression. Cu levels increasing induces an increase in mRNA linked to VEGF production and simultaneously induces the maturation of hypoximirs. COMMD1 plays a key role in this cascade mechanism, as its deletion has been shown to reduce Cu loss after hypoxic injury.

A recent study on bio-organometallics showed that the Cu complex can induce an increase in cystathionine γ-lyase (CSE) mRNA in rat aortic endothelial cells, an oxidative stress protective enzyme involved in reactive sulfur species synthesis. The hypoxic condition causes the expression of HIF-1α to increase but does not change the levels of HIF-1α-associated mRNA. The siRNA-mediated knockdown of HIF-1α or HIF-1β partially suppressed CSE transcription in endothelial cells induced by Cu10 (Figure 3). These findings suggest that a Cu complex (Cu10) can induce HIF-1α/HIF-1β hypoxia signaling in a process mediated by CSE [130].

Li and collaborators showed that the coronary artery ligation caused an ischemic injury both in the WT and COMMD1 knockout mice, and 7 days after HF, the Cu concentrations decreased 2.8-fold in WT mice but 1.7-fold in the COMMD1 knockout mice. The serum Cu concentrations increased in both cases. The tissue’s Cu loss induced cardiac dysfunction. Interestingly, in the COMMD1 knockout mice, the myocardial dysfunction was much less severe than that in the WT mice, suggesting that COMMD1 deletion preserved myocardial contractile function and reduced the infarct size. Seven days after the injury, the COMMD1 knockout mice showed a significant increase in capillary density compared with the WT mice due to the conservation of VEGF and VEGFR-1 mRNA levels. Conversely, the knockout mice presenting the deletion of COMMD1 had no significant changes related to HIF-1a expression [111]. Cu depletion was caused by myocardial ischemia [131], and in Li’s study, the authors demonstrated that COMMD1 deletion prevents Cu loss in the cardiomyocyte after HF, suggesting a protective effect of the Cu balance on the heart mediated by Cu retention, which was associated with the partial preservation of cardiac contractile function.

Interestingly, a direct interaction between COMMD1 and HIF-1a in ischemic injury was found. It has been shown that in mouse embryos, COMMD1 knockout increased HIF-1α stability, even though overexpression of COMMD1 in human cell lines inhibited HIF-1 activity [132]. Conversely, the cardiomyocyte-specific deletion of COMMD1 preserved the Cu efflux in the heart and induced some critical biological processes such as HIF-1 transcriptional activity. After ischemic insult, the Cu and HIF-1 preserved the cardiac structure and function [94].

In the future, it could be important to understand how ischemia causes COMMD1 elevation in the heart to understand if the expression of some hypoxia-induced miRNA, especially the miR-200 family, can have an effect on COMMD1 function in order to define their exact role in the Cu, COMMD1, and HIF-1 pathways.

## 4. Conclusions

The role of the miRNAs in AD has not yet been clarified, and the literature collected so far reports controversial results, as is the case for miR-200b, which has been shown to be downregulated in some studies and upregulated in others.

A recent meta-analysis [101] highlighted the difficulties encountered when studying miRNAs in AD patients. Of about 1500 miRNAs extracted from different biological matrices (blood, CFS, MCI, and the brain), 56 miRNAs showing significant differential expression in AD compared with the healthy controls and 10 novel miRNAs were found in the blood, and 2 of them were downregulated: miR-103, which is involved in neurite transportation, and miR-107, which is involved in BACE1 regulation. miRNAs that could be therapeutic candidates for the treatment of AD are miR-15a-5p, miR-103a-3p, and miR-29a-3p because they are associated with Aβ clearance, autophagic processes, inflammation, and toxicity.

In mouse models of MI induced by experimental ischemia, hypoxia has been associated with Cu dysfunction, featuring the release of Cu from the heart to the bloodstream up to 80% and mediated by the increased activity of the Cu transporter COMMD1. It could be hypothesized that there is a link between Cu dynamics following MI and the induction of miRNAs induced by hypoxia linked to CVD and AD. A possible candidate could be miR-107, which is involved in BACE1 activity. For these reasons, it could be strategic to investigate the intertwining interactions of microRNAs and HIF-1 in the control of Cu’s balance in AD and in CVD.

## Data Availability

The study did not report any data.
